# CircRNA ANXA2 Promotes Lung Cancer Proliferation and Metastasis by Upregulating PDPK1 Expression

**DOI:** 10.1155/2021/4526609

**Published:** 2021-12-28

**Authors:** Yan Ju, Bin Yuan, Wenan Wu, Jin Zhao, Xiaorui Shi

**Affiliations:** Combine Traditional Chinese and Western Medicine, Shaanxi Cancer Hospital, Xi'an, Shaanxi 710061, China

## Abstract

Lung cancer is a common malignant tumor that seriously threatens human health. It has become the top malignant tumor in terms of morbidity and mortality. In recent years, circRNA, a special noncoding RNA molecule, has attracted considerable interest. This study focused on the role of circRNA ANXA2 (circANXA2) in lung cancer and the molecular mechanism of cancer promotion. Real-time quantitative PCR (RT-PCR) was used in detecting the expression abundance of circANXA2 in different lung cancer cells and tissues. The subcellular localization of circANXA2 was detected through fluorescence in situ hybridization. circANXA2 expression was knocked down through siRNA. CCK-8, clone formation assay, and TUNEL assay were used in evaluating the effects of circANXA2 on cell proliferation, clone formation ability, and apoptosis. The role of circANXA2 in tumor proliferation was further verified in vivo using the tumor transplantation model in nude mice. The molecular mechanism of circANXA2 was investigated with luciferase activity assay and RT-PCR. The expression abundance of circANXA2 is high in lung cancer cell lines and tissues. Knocking down of circANXA2 inhibits the proliferation and clonogenesis of the lung cancer cells. Knocking down circANXA2 promotes apoptosis. circANXA2 further affects downstream PDPK1 expression by regulating miR-33a-5p and thereby affecting the malignancy of the lung cancer cells. circANXA2 inhibits miR-33a-5p activity by directly interacting with miR-33a-5p. circANXA2 regulates the transcription of the miR-33a-5p downstream target gene PDPK1 and affects the malignant progression of lung cancer.

## 1. Introduction

Lung cancer is the leading cause of tumor-related death [1], and the high morbidity and mortality of lung cancer have imposed heavy social and economic burdens [2]. The key to preventing and treating lung adenocarcinoma is exploring the mechanism of the occurrence and development of lung cancer from the aspects of gene regulation and finding novel early diagnosis methods and therapeutic targets [3–5].

Noncoding RNAs play an important role in tumor development and form complex regulatory networks [6, 7]. circRNAs are noncoding RNA molecules formed end to end during transcription [8] and play important roles in many pathophysiological processes [9]. circRNAs can affect the occurrence and development of a variety of tumors, including lung cancer, through mechanisms, such competitive microRNA inhibition [10]. Although some progress has been made in the study of circRNAs, understanding about the role of circRNAs in tumors is still in its infancy. Hence, the role and function of circRNAs in tumors need to be further studied, as well as the downstream regulation mechanism and target genes of circRNAs. circANXA2 is involved in tumor genesis and progression. Zhang et al. demonstrated that circANXA2 is a potential biomarker and therapeutic target for AML by analyzing circRNA profiles [11]. However, the role of circANXA2 in lung cancer has not been reported.

Mature miRNAs recognize target mRNAs through base complementary pairing [12]. According to the degree of complementarity, miRNAs induce the silencing complex to degrade target mRNAs or block the translation of target mRNAs [13]. miR-33a is involved in a variety of physiological and pathological processes, including metabolism, cell differentiation, tumor genesis, and development [14]. In breast cancer [15] and osteosarcoma [16], miR-33a acts as a tumor suppressor by targeting multiple oncogenes. In lung cancer cells, the role of miR-33a needs to be further studied.

The 3-phosphoinositide-dependent kinase 1 (PDPK1) structure is composed of a 63 kDa serine/threonine (Ser/Thr) protease [17]. PDPK1 is an upstream activated kinase of Akt1 [18, 19]. The C-terminal pH region is compatible with phosphatidylinostol-3,4,5,-triphosphate (Pi2P3) and the phosphorylates Akt/PKB. The Pi2P3-dependent protein kinase PDPK1 binds to Pi2P3 through its own pH domain in the cytoplasm, causing conformational changes in Akt and PDPK1 to be close to each other. PDPK1 can activate many other proteins, such as some PKC subtypes and some members of the AGC family, including p70 ribosomal S6 kinase (S6K), P90 ribosomal S6 kinase (RSK), serum and glucocorticoid-induced kinases (SGKs), and protein kinase A (PKA) [20, 21]. PDPK1-mediated PI3K/Akt signaling is associated with many types of cancers [22, 23]. However, the role of PDPK1 in lung cancer is not well understood.

In this study, we first evaluated the expression level of circANXA2 in lung cancer and paracancerous tissues at the clinical level. The effects of downregulated circANXA2 expression on the proliferation, apoptosis, and cycle of lung adenocarcinoma cells were further observed with cell function experiments. Possible miRNA targets and the downstream genes of circANXA2 were detected using bioinformatics methods and dual luciferase reporter assay. The regulatory mechanism of ceRNA constituted by circANXA2 was finally confirmed. The role and mechanism of circANXA2 in lung adenocarcinoma were preliminarily explored, and the role of circANXA2 in the regulatory network of lung adenocarcinoma was clarified. The primary aim was to find novel early diagnostic markers and therapeutic targets.

## 2. Methods

### 2.1. Clinical Sample Collection and Patient Information Collection

Lung adenocarcinoma tissue samples used in this study were all collected from patients who had a definite diagnosis of lung adenocarcinoma and underwent surgery in the thoracic surgery department of our hospital between September 2020 and February 2021. Lung cancer and adjacent control tissues from 20 patients were collected. Patient inclusion criteria were as follows: patients with lung adenocarcinoma who underwent lobectomy in the thoracic surgery department of our hospital; imaging findings supported the diagnosis of lung adenocarcinoma; pathological diagnosis was lung adenocarcinoma; preoperative radiotherapy, chemotherapy, targeted drug therapy, and biological therapy were not received; and agreed to participate in the study and signed the informed consent form. The exclusion criteria were as follows: complicated or suspected with other chronic respiratory diseases, such as COPD and bronchiectasis; routine pathological diagnosis after surgery failed to confirm lung adenocarcinoma; and other underlying diseases that may have interfered with research. Pathological diagnosis was made independently by two senior pathologists. The clinical information of patients was recorded completely before surgery. Samples of lung adenocarcinoma and normal lung tissues that were more than 5 cm away from the lung adenocarcinoma foci were collected during the operation, placed in RNA-free cryopreservation tubes within 10 min, and rapidly cooled with liquid nitrogen. Under low-temperature conditions, the samples were transferred to the −80°C deep low-temperature refrigerator. All patients signed informed consent forms. This study was reviewed and approved by the Ethics Committee of Shaanxi Cancer Hospital and in line with the Declaration of Helsinki (IRB-SC-2020-012).

### 2.2. Cell Culture

Human lung epithelial cell BEAS-2B and lung cancer cell lines A549, NCI-H446, NCI-H460, PC-9, and 95-D were all purchased from the Institute of Biochemistry and Cell Biology, Chinese Academy of Sciences, and processed in our laboratory. The cells were amplified in the high-glucose DMEM culture medium containing 10% fetal bovine serum (Gibco, Life Technologies, Rockville, MD, USA). In this study, the function of circANXA2 was analyzed in the A549 and PC-9 cell lines. A549 cells belong to human nonsmall cell lung cancer cells and constructed from the lung cancer tissue transplantation culture by D. J. Griad. The cells were obtained from a 58-year-old white male [24, 25]. A549 can synthesize lecithin, which contains a high amount of unsaturated fatty acids, through the cytidine diphosphate choline pathway. A549 cells are positive for keratin. PC-9 is a human nonsmall cell lung cancer cell with EGFR 19 exon mutation [26, 27]. Cells were cultured in an adherent cell culture flask or culture plate, and cell growth was observed daily. The medium was replaced every 2–4 days according to the cell density. The cells were cultured in a 37°C, 5% CO_2_ wet constant temperature incubator (Thermo Fisher Scientific, Waltham, MA, USA). Once the cell density reached 80%–90%, cell passage and cryopreservation were carried out according to the experimental needs.

### 2.3. Cell Transfection

Cell growth was observed, and the cells were digested after an optimal passage time was reached. The cells were seeded in six-well cell culture plates at a density of 2.5 × 10^5^/well. The culture was carried out in a complete medium, and cell growth was observed 24 h later. Liposome transfection experiments were carried out after the degree of cell confluence reached 60%–70%. At least 20 min before the experiment, the original medium was discarded and replaced with the serum-free DMEM medium (1.5 mL/well). Prepare for Lipofectamine 2000 transfection (Life Technologies, Rockville, MD, USA). The effect of siRNA degradation was prevented by conducting the whole process of liposome transfection in an environment without RNA enzymes. According to the purpose of the study and different siRNAs transfected, experimental groups were set. The details were as follows: (1) si-circANXA2 transfection group (si-circRNA group): circANXA2 siRNA; (2) siRNA transfection group (si-NC group): random siRNA transfection; (3) blank control group: only liposomes were added and siRNA transfection was not performed. The liposome transfection procedure was carried out according to the manual of Lipofectamine 2000.

### 2.4. Gene Expression Was Detected by qRT-PCR

Total RNA was extracted by the TRIzol (Sigma-Aldrich, St. Louis, MO, USA) method according to the kit's technical manual. The purity and concentration of total RNA were detected using a NanoDrop 1000 spectrophotometer (Thermo Scientific, USA). The total RNA of the cell was converted into cDNA with a reverse transcription kit. The cDNA of a lung adenocarcinoma tissue or cell was used as template, and the upstream and downstream primers of the target gene were used. Possible reverse transcriptome fragments were amplified through PCR reactions. Each sample was equipped with three duplicate holes to improve the reliability of the results. RT-PCR was performed for reaction and detection. The reaction process and conditions were as follows: circANXA2, forward: 5′-ACCTGCTCAGTATGACGCTTCT-3′, reverse: 5′-CTGGTAGGCGAAGGCAATATCC-3′; miR-33a-5p, forward: 5′-GGTGCATTGTAGTTGCATTGC-3′, reverse: 5′-GTGCAGGGTCCGAGGTATTC-3′. E-cadherin, forward: 5′-TGCCCAGAAAATGAAAAAGG-3′, reverse: 5′-GTGTATGTGGCAATGCGTTC-3′; vimentin forward: 5′-GAGAACTTTGCCGTTGAAGC-3ʹ, Reverse: 5ʹ-GCTTCCTGTAGGTGGCAATC-3ʹ. PDPK1, forward: 5′-GGAACAGCGCAGTACGTTTCT-3′, reverse: 5′-CTCGTTTCCAGCTCGGAATGG-3′; U6: forward: 5′-ATTGGAACGATACAGAGAAGATT-3′, reverse: 5′-GGAACGCTTCACGAATTTG-3′; GAPDH, forward: 5′-AAGCCTGCCGGTGACTAAC-3′, reverse: 5′-GCGCCCAATACGACCAAATC-3′. The PCR reaction conditions were as follows: predenaturation at 95°C for 3 min; 35 cycles of 95°C denaturation for 35 s, 53°C annealing for 35 s, and 72°C extension for 40 s; and extension at 72°C for 10 min. The reaction was stopped at 10°C. Sequence information: CT values of measured tissue and cell samples and internal reference GAPDH were recorded, and the average CT value from the three reholes was obtained for each sample. The 2^△△Ct^ values were used to indicate the relative expression levels of the target gene in the lung adenocarcinoma tissues.

### 2.5. Western Blot

Total cell protein was extracted from transfected cells with a cell protein extraction kit. Separation was performed on a 10% SDS-PAGE gel, and the cells were transferred to a PVDF (Millipore, Billerica, MA, USA) membrane. The membrane containing 5% skim milk was closed at room temperature for 1 h. The first antibody was used overnight at 4°C. Finally, these membranes were incubated with secondary antibodies and developed.

### 2.6. Experiment of Fluorescent In Situ Hybridization

The cells were digested and counted. After the cell slide was placed, the number of cells added to each hole was approximately 5 × 10^4^. The cell density reached approximately 50%–60%. 1 × PBS was added to each well, and the cells were washed for 5 min. Then, 500 *μ*L of 4% paraformaldehyde was added to each well. The cells were left at room temperature for 10 min. Waste liquid was disposed, and 1 mL of permeable liquid was added at 4°C to each operating hole for 5 min. A prehybridization solution was prepared, and proper amounts of blocking solution and prehybridization buffer were mixed in a centrifugal tube in a ratio of 1 : 99. The prehybrid solution (200 *μ*L) was added to each well and reacted at 37°C for 30 min. In the dark, 2.5 *μ*L of FISH Probe Mix with a concentration of 20 *μ*M was added at 37°C overnight. Then, cleaning fluid was added. DAPI staining solution was diluted in a ratio of 1 : 1000 under light protection, and 200 *μ*L of the solution was added to each well for dyeing for 10 min. The cell slides were carefully removed (away from light) and fixed on glass slides with a sealing tablet. Fluorescence detection was carried out, and photographs were taken.

### 2.7. CCK-8

The cell suspension was diluted with a complete medium for the adjustment of cell density to 5 × 10^4^/mL. The suspension was inoculated at 100 *μ*L/well on 96-well cell culture plates. After 24 h of culture, the degree of confluence was observed, and cell transfection was carried out when the confluence was approximately 70%. Five duplicate wells were set in each group, and cell-free wells were set as references. CCK-8 (Beyotime, Shanghai, China) detection solution (10 *μ*L) was added to each well 72 h after transfection, and the cells were placed in an incubator for 4 h. The absorbance value (OD value) of each well at 450 nm was determined using a microplate analyzer. The above experiment was performed three times.

### 2.8. Clone Formation Experiment

Six-well plates were prepared, and 2 mL of the preheated DMEM complete medium and 100 *μ*L of cell suspension (containing about 200 cells) were added to each well at 37°C. Liquid was changed every 3-4 days, and cultivation was performed for 2 weeks. The culture was stopped when the clones were visible to the naked eye at the plates. After the culture was stopped, the supernatant of each hole was extracted, and the holes were cleaned with PBS twice. Paraformaldehyde (4%) was added to fix the cells for approximately 10 min. The stationary solution was discarded, and the cells were washed carefully with PBS twice. Treatment with 0.1% crystal violet (Beyotime, Shanghai, China) staining solution was performed for 10 min, and the staining solution was washed off with PBS. The wells were dried at room temperature. The number of clones formed was counted under a microscope, recorded, and photographed.

### 2.9. TUNEL Experiment

Cells in the logarithmic growth phase were extracted, the concentration of cells was adjusted to 1 × 10^5^, and the cells were seeded into cell slides. Each specimen was rinsed three times with PBS. After drying in air, the samples were fixed with freshly prepared, and a paraformaldehyde solution was added for 1 h at room temperature. After the samples were washed with PBS, treatment with 0.03% H_2_O_2_ was performed at room temperature for 10 min for the removal of endogenous peroxidase activity. The film was broken with 0.1% Triton X-100. Treatment was performed at 4°C for 2 min. Finally, after the samples were dried, they were rinsed with PBS. Reaction mixtures (2 *μ*L of enzyme concentrate and 18 *μ*L of labeled solution) and deoxynucleotide transferase solution were added. The samples were incubated at 37°C in the 5% CO_2_ incubator for 60 min. After DAPI staining, the samples were observed under a fluorescence microscope.

### 2.10. Double Luciferase Reporter Gene Assay

The full-length sequences of circANXA2 and PDPK1 3′-UTR with and without the miR-33a-5p mutant binding site were cloned. The downstream of Renilla luciferase gene cloned into the double plasmid psiCHECK2 vector (Promega, Madison, WI, USA). The psiCHECK2-circANXA2 and psiCHECK2-PDPK1-3′-UTR vectors (Promega) were constructed. Subsequently, 10 pmol miR-33a-5p and control mimics were cotransfected with luciferase vectors. The dose of the transfection reagent was 40 ng of Lipofectamine 3000. Luciferase activity was measured with a luciferase report assay kit (Promega) 48 h after transfection. All experiments were repeated three times.

### 2.11. Subcutaneous Tumor Bearing Test

After the nude mice were fed for a week, subcutaneous lung cancer tumor transplantation models were established, and each group contained 6 nude mice. Alcohol was used to disinfect the back skin of the nude mice, and 0.2 mL of the prepared lung cancer A549 cell (Lenti-sh-NC, Lenti-sh-circANXA2) suspension was extracted with a syringe and inoculated subcutaneously on the back of the nude mice. After the tumor cells were inoculated, the epidermis was visible at the skin injection site on the back. After 1 week, the diameter of the tumor grew to 5-6 mm, indicating the successful establishment of a subcutaneous tumor transplantation model in nude mice. Naked mice with transplanted and metastatic tumor models were fed in an SPF environment. The diet and mental states of the nude mice were observed regularly. The weights of the mice were recorded every week. The long diameter (*L*) and short diameter (*W*) perpendicular to the subcutaneous graft tumor were measured. Tumor volume (*V*) was calculated using formula *V* = (*L* × *W*^2^)/2, and a tumor growth curve was plotted. After the experiment, the nude mice were sacrificed through the dislocation of the neck. Tumor mass was extracted and weighed. Within half an hour of tissue isolation, a part of the mass was preserved with liquid nitrogen, and another part was fixed in paraformaldehyde. Tumor tissue was removed for immunohistochemical detection. Animal experiments were approved by the Ethics Committee of Shaanxi Cancer Hospital.

### 2.12. Immunohistochemical Staining

The tissue was cut into 4 *μ*m thick slices and baked in an oven at 60°C for half an hour. The wax was dewaxed with xylene 3 times for 5 min each. Treatment was performed with anhydrous ethanol twice for 5 min each. The tissues were soaked in 95% ethanol twice for 5 min each. Distilled water was used 2 times for 5 min each and closed with 3% hydrogen peroxide for 15 min. After the sections were removed, they were washed with PBS 3 times for 5 min each. Normal goat serum (10%) was sealed for 30 min. The primary antibody was added dropwise, and the samples were incubated overnight at 4°C. The primary antibody was removed, a polymer-reinforcing agent was added, and the mixture was balanced at room temperature for 30 min. The second antibody was added, and the samples were incubated at 37°C for 60 min. DAB color and color intensity under the microscope were controlled. After the color is finished, it was placed in distilled water and dehydrated with ethanol according to the concentration gradient. Transparent treatment was performed with xylene, and neutral gum was used for sealing.

### 2.13. Statistical Analysis

SPSS 17.0 software (SPSS Inc., Chicago, IL, USA) was used for the statistical analysis of the experimental results. Measurement data were expressed as mean ± standard deviation. Independent sample Student's *t*-test was used for comparison between the two groups. The mean values of the groups were compared with one-way analysis of variance followed by Tukey's post hoc test. Counting data were tested with the chi-square test. A *P* value of <0.05 was considered statistically significant.

## 3. Results

### 3.1. Detection of circANXA2 Expression Levels in Lung Adenocarcinoma Tissues and Cells

**Figure 1:**
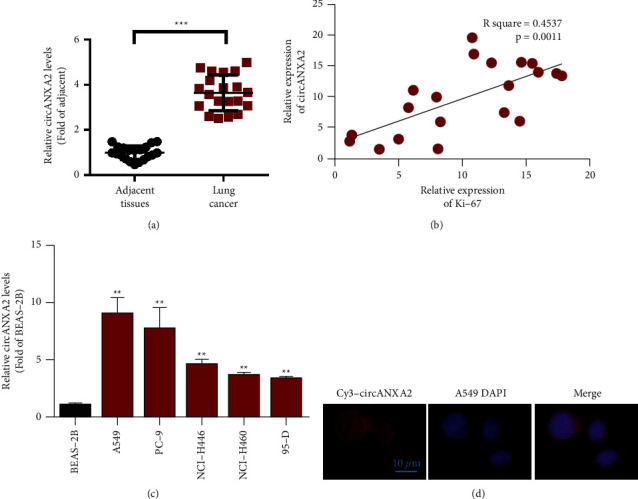
circANXA2 is upregulated in lung cancer tissues and is related to the prognosis of lung cancer patients. (a) qRT-PCR was used to detect the expression of circANXA2 in tumor tissues and adjacent tissues of lung cancer patients. (b) Ki-67 is positively correlated with circANXA2. (c) CircANXA2 is upregulated in lung cancer cell lines. (d) FISH assay was used to detect the expression and location of circANXA2 in cells. ^∗^*P* <0.05. ^*∗∗*^*P* < 0.01. ^*∗∗∗*^*P* < 0.001.

Surgical specimens from patients with lung adenocarcinoma were detected through qRT-PCR. The results showed that the expression of circANXA2 was significantly upregulated in the lung adenocarcinoma tissues compared with that in the adjacent tissues, with statistical significance (Figure 1(a)). The detection of coexpression correlation between circANXA2 and proliferation marker Ki-67 showed that the coexpression levels of circANXA2 and Ki-67 were positively correlated (Figure 1(b)). Subsequently, we detected the expression abundance of circANXA2 in human normal lung epithelial cells (BEAS-2B) and in different lung cancer cells. Furthermore, the cell lines of circANXA2 specifically highly expressed in lung cancer were identified. As shown in Figure 1(c), the expression levels of circANXA2 lung cancer cell lines were significantly higher than those in the BEAS-2B cells. Fluorescent in situ hybridization (FISH) is a new in situ hybridization method based on fluorescence labeling instead of isotope labeling and has many advantages, such as safety, rapidness, high sensitivity, and probes with long preservation times. We used FISH to detect the sublocalization of circANXA2 in the lung cancer cells. As shown in Figure 1(d), circANXA2 was mainly localized in the cytoplasm of the lung cancer cells.

### 3.2. Effects of Knocking Down circANXA2 on the Proliferation, Clone Formation, and Apoptosis of Lung Cancer Cells

**Figure 2:**
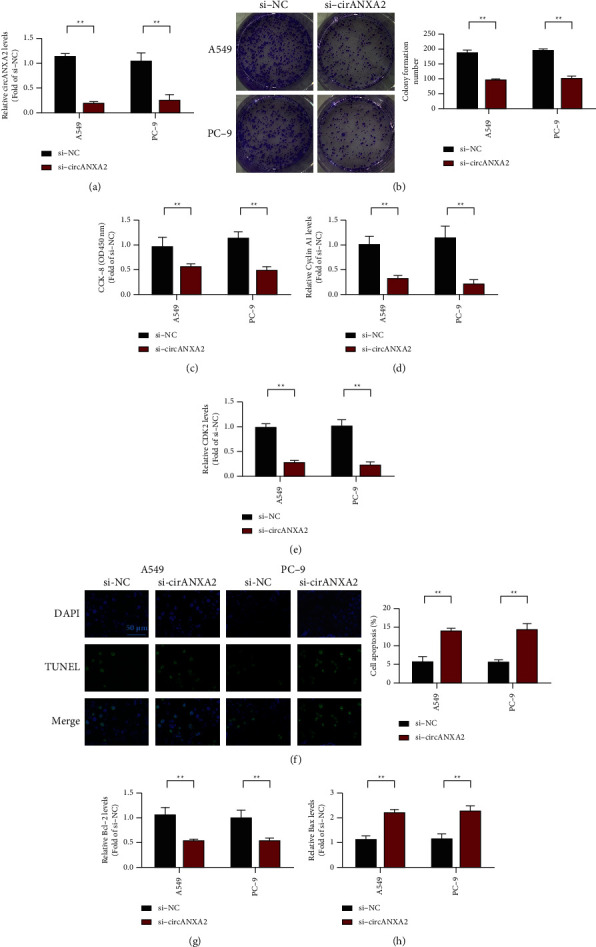
Cell level proves that knocking down circANXA2 inhibits the malignant behavior of lung cancer cells. (a) Detection of the expression level of circANXA2 in A549 and PC-9 cells. (b) The effect of circANXA2 on the cloning ability of A549 and PC-9 was tested. (c) CCK-8 detects the effect of circANXA2 on the proliferation of A549 and PC-9. (d) After knocking down circANXA2, the expression of cyclin A1 in A549 and PC-9 cells was detected. (e) After knocking down circANXA2, the expression of CDK2 in A549 and PC-9 cells was detected. (f) TUNEL detects the effect of circANXA2 on A549 and PC-9 cell apoptosis. (g) After knocking down circANXA2, the expression of Bcl-2 in A549 and PC-9 cells was detected. (h) After knocking down circANXA2, the expression of Bax in A549 and PC-9 cells was detected. ^*∗∗*^*P* < 0.01.

The proliferation of tumor cells can reflect the degree of malignancy. The rate of tumor proliferation increases with the degree of malignancy, but doubling time is shortened. CCK-8 was used in detecting the proliferation of A549 and PC-9 cells after circANXA2 knockdown.  Figure 2(a) shows the knockout efficiency of siRNA. circANXA2 siRNA significantly reduced the expression of circANXA2 (Figure 2(a)). The effect of circANXA2 on the clone formation ability of A549 and PC-9 was detected. After knockdown of circANXA2, the clonogenic ability of A549 and PC-9 cells was significantly inhibited, suggesting that the knockdown of circANXA2 can inhibit the clonogenic ability of lung cancer cells (Figure 2(b)). circANXA2 knockdown significantly inhibited the growth of A549 and PC-9 cells, suggesting that circANXA2 knockdown can inhibit the proliferation of small cell lung cancer (Figure 2(c)). Subsequently, we detected changes in the expression levels of proliferation-related proteins cyclin A1 and CDK2. The experimental results showed that the expression levels of cyclin A1 and CDK2 decreased after circANXA2 knockdown (Figures 2(d) and 2(e)). To determine whether circANXA2 affects the apoptosis of lung cancer cells, was performed TUNEL staining to detect it. TUNEL fluorescence analysis was performed in the control and si-circANXA2 groups. The results showed that the apoptosis of A549 and PC-9 cells was significantly increased after circANXA2 knockdown (Figure 2(f)). The Bcl-2 expression level detection results showed that after circANXA2 was knocked down, Bcl-2 expression decreased (Figure 2(g)). However, after circANXA2 was knocked down, the expression of Bax was upregulated (Figure 2(h)).

### 3.3. circANXA2 Knockdown Inhibits the Proliferation of Lung Cancer Cells

**Figure 3:**
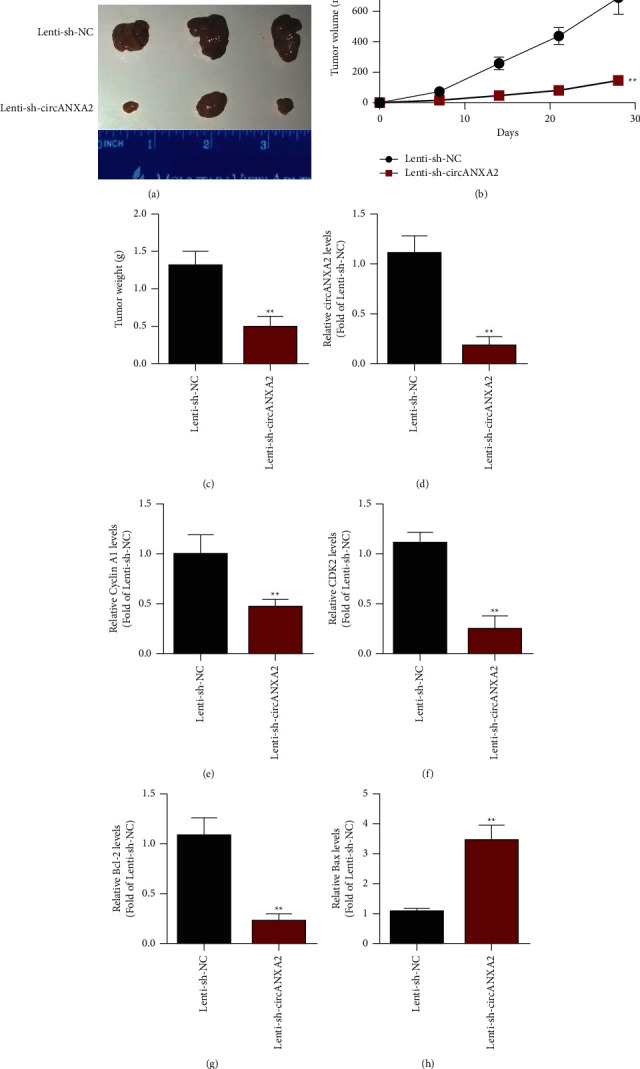
Subcutaneous tumor-bearing experiments in nude mice proved that knocking down circANXA2 inhibits the proliferation of lung cancer cells. (a) Tumor growth curve of nude mice. *N* = 6. (b) Results of nude mouse tumor. (c) Tumor weight of nude mice. (d) Detection of the expression level of circANXA2 in two groups of tumor tissues. (e) After knocking down circANXA2, the expression of cyclin A1 in tumor tissues was detected. (f) After knocking down circANXA2, the expression of CDK2 in tumor tissues was detected. (g) After knocking down circANXA2, the expression of Bcl-2 in tumor tissues was detected. (h) After knocking down circANXA2, the expression of Bax in tumor tissues was detected. ^*∗∗*^*P* < 0.01.

In the cell level studies of circANXA2, we showed that the knockdown of circANXA2 reduces the malignancy of lung cancer cells. We confirmed whether circANXA2 has a tumor-suppressive effect in vivo, using the nude mouse tumor transplantation model. In this in vivo experiment, BALB/c nude mice with immune deficiency of 4–6 weeks were selected for the establishment of an animal model. Two groups (Lenti-sh-NC and Lenti-sh-circANXA2) were established, and each group had six nude mice. Animal experimental results showed that the proliferation rates of tumor tissues in the nude mice in the sh-circANXA2 group decreased and tumor volumes were small (Figures 3(a) and 3(b)). The tumor weights of the nude mice in the SH-circANXA2 group were lower than those in the control group (Figure 3(c)). The expression of circANXA2 in subcutaneous tumor tissues was detected with qRT-PCR. The expression of circANXA2 decreased in the tumor tissues of the SH-circANXA2 group (Figure 3(d)). The expression levels of cyclin A1 and CDK2 in the tumor tissues of the models were detected with qRT-PCR. The expression levels of cyclin A1 and CDK2 decreased in the sh-circANXA2 group (Figures 3(e) and 3(f)). In addition, the expression of Bcl-2 in tumor tissues decreased after circANXA2 knockdown (Figure 3(g)). The expression of Bax was upregulated (Figure 3(h)).

### 3.4. circANXA2 Can Bind to miR-33a-5p

**Figure 4:**
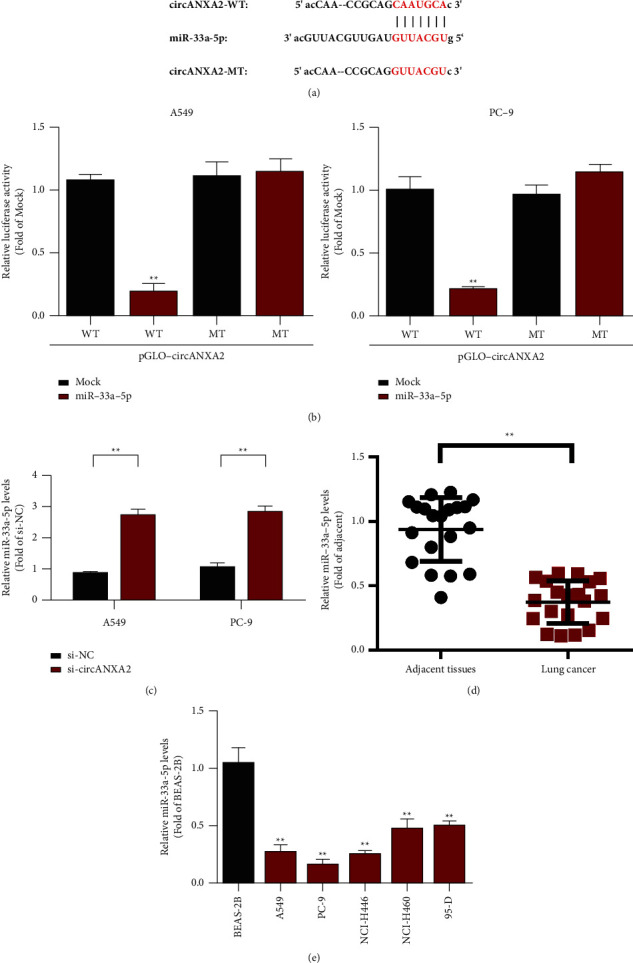
circANXA2 can act as a sponge for miR-33a-5p in lung cancer cells. (a) CircANXA2 and miR-33a-5p binding site displayed. (b) Report carrier experiment. (c) Detection of the expression level of miR-33a-5p by circANXA2 in A549 and PC-9 cells. (d) Detection of the expression level of miR-33a-5p in adjacent and lung cancer tissues. (e) The expression of miR-33a-5p is downregulated in lung cancer cell lines. ^*∗∗*^*P* < 0.01.

We further screened miRNAs that may bind to circANXA2. We first used the bioinformatics analysis method. The prediction results showed that circANXA2 may bind to miR-33a-5p (Figure 4(a)). To further verify the relationship between circANXA2 and miR-33a-5p, we constructed a dual luciferase reporter gene detection system. We cloned the linear sequence of circANXA2 downstream of the luciferase gene. As the 3′UTR region of the luciferase gene, if miR-33a-5p can be bind to this circRNA, it is equivalent to acting on the 3′UTR region of the luciferase gene and inhibiting its normal transcription. In this experiment, the constructed luciferase gene was transferred to cells together with the luciferase gene of the sea kidney. MOCK and miR-33a-5p mimics were transferred into 293T cells successively. The cells were collected after 48 h, and luciferase activity was detected. The results showed that luciferase activity in the circANXA2 wild-type group was significantly lower in the miR-33a-5p transferring group than in the MOCK group. However, in the circANXA2 mutant group, luciferase activity in the miR-33a-5p transferring group did not significantly change compared with that in the MOCK group (Figure 4(b). In A549 and PC-9 cells, the expression level of miR-33a-5p increased after circANXA2 knockdown (Figure 4(c)). The surgical specimens of patients with lung adenocarcinoma were detected with qRT-PCR, and the results showed that the expression of miR-33a-5p in lung adenocarcinoma tissues was significantly downregulated compared with that in adjacent tissues, with statistical significance (Figure 4(d)). Further experimental results showed that miR-33a-5p was downregulated in the lung cancer cell lines compared with that in the human normal lung epithelial cell BEAS-2B (Figure 4(e)).

### 3.5. Overexpression of miR-33a-5p Reverses the Promoting Effect of circANXA2 on Lung Cancer Cells

**Figure 5:**
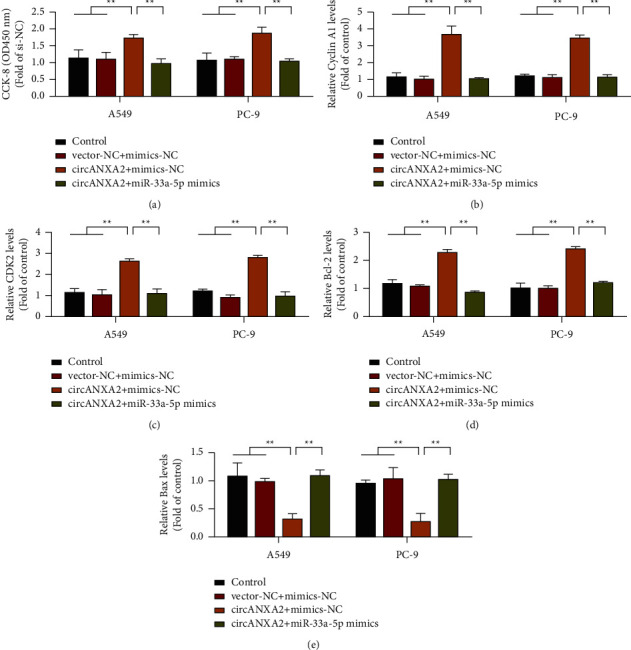
Overexpression of miR-33a-5p reverses the promoting effect of circANXA2 on lung cancer cells. (a) CCK-8 verifies that miR-33a-5p in A549 and PC-9 cells reverse the effect of circANXA2 on cell proliferation. (b) Detection of cyclin A1 expression in A549 and PC-9 cells. (c) Detection of CDK2 expression in A549 and PC-9 cells. (d) Detection of Bcl-2 expression in A549 and PC-9 cells. (e) Detection of Bax expression in A549 and PC-9 cells. ^*∗∗*^*P* < 0.01.

The binding ability of miR-33a-5p to circANXA2 was confirmed using the luciferase reporter gene technique described above. However, further functional experiments are needed to verify whether the effect of circANXA2 on the malignancy of lung cancer occurs through miR-33a-5p. In this study, we simultaneously transfected miR-33a-5p mimics into lung cancer cells that overexpressed circANXA2. Mimics-NC was used as a negative control. Whether the overexpression of miR-33a-5p can reverse the cancer-promoting effect of circANXA2 was determined. The results of CCK-8 detection are shown in Figure 5(a). The overexpression of circANXA2 promoted the proliferation of A549 and PC-9 cells, but the proliferation of lung cancer cells decreased after the addition of miR-33a-5p mimics. Furthermore, the overexpression of circANXA2 upregulated the expression levels of cyclin A1, CDK2, and Bcl-2. However, after the addition of miR-33a-5p mimics, the expression levels decreased (Figures 5(b)–5(d)). The expression of Bax was inhibited by circANXA2 overexpression, but the expression level increased after the addition of miR-33a-5p mimics (Figure 5(e)). These results suggest that circANXA2 may regulate the function of its downstream genes by binding with miR-33a-5p.

### 3.6. miR-125a-5p Targeted PDPK1 Binding

**Figure 6:**
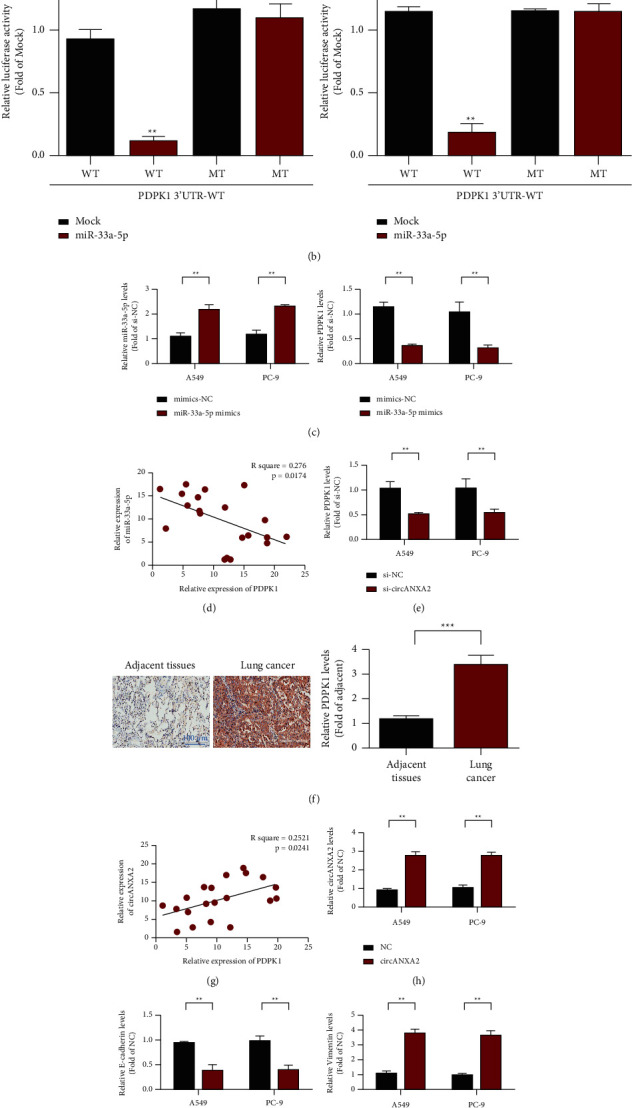
MiR-125a-5p targets PDPK1. (a) PDPK1 locus information for miR-33a-5p targets. (b) Report carrier experiment. (c) qRT-PCR detects that miR-33a-5p inhibits the expression of PDPK1. (d) Correlation analysis of coexpression of miR-33a-5p and PDPK1. (e) qRT-PCR detection knockdown circANXA2 inhibits the expression of PDPK1. (f) Immunohistochemical detection of PDPK1 expression levels in adjacent and lung cancer tissues. (g) The coexpression correlation between circANXA2 and PDPK1. (h) circANXA2 transfection efficiency test results. (i) Effect of circANXA2 overexpression on E-cadherin expression in A549 and PC-9 cells. (j) The effect of circANXA2 overexpression on vimentin expression in A549 and PC-9 cells. ^*∗∗*^*P* < 0.01.

The experiments showed that circANXA2 can bind to miR-33a-5p, but whether circANXA2 can regulate the expression of its downstream target genes through miR-33a-5p remains unclear. Using the TargetScan website, we further predicted that miR-33a-5p can bind to the 3′UTR region of the PDPK1 gene in lung cancer cells (Figure 6(a)). The dual luciferase reporter gene assay further confirmed that miR-33a-5p can bind to PDPK1. In the PDPK1 wild-type group, luciferase activity in the miR-33a-5p transferring group was significantly lower than that in the MOCK group. However, in the PDPK1 mutant group, luciferase activity in the miR-33a-5p transferring group did not significantly change compared with that in the MOCK group (Figure 6(b)). The qRT-PCR results showed that the expression of PDPK1 decreased after the overexpression of miR-33a-5p (Figure 6(c)). Correlation analysis results of the coexpression of miR-33a-5p and PDPK1 showed that the coexpression levels of miR-33a-5p and PDPK1 were negatively correlated (Figure 6(d)). After circANXA2 was overexpressed, the expression of PDPK1 gene was detected. The expression of the PDPK1 gene was significantly increased after circANXA2 overexpression, as shown in Figure 6(e). Immunohistochemical results showed that PDPK1 was highly expressed in the lung cancer tissues (Figure 6(f)). We further analyzed the coexpression correlation between circANXA2 and PDPK1. The results showed that circANXA2 and PDPK1 had positive coexpression correlation (Figure 6(g)). The results of the analysis of the influence of overexpression of circANXA2 on the expression levels of epithelial marker E-cadherin and mesenchymal marker vimentin in lung cancer cells showed that overexpression of circANXA2 inhibited the expression of E-cadherin but upregulated vimentin. These results indicate that circANXA2 can promote EMT in lung cancer cells (Figures 6(h)–6(j)).

## 4. Discussion

Lung cancer is one of the leading causes of cancer-related death worldwide and can be divided into nonsmall cell lung cancer (NSCLC) and small cell lung cancer (SCLC) [28, 29].

At present, the role of circRNA “sponges” that adsorb miRNAs is the most widely studied [30]. CircGFRA1 is highly expressed in NSCLC tissues and regulates the proliferation of NSCLC cells by binding to miR-183-3p and regulating the PIK3/Akt signaling pathway [31]. Hsa_circ_0012673 is overexpressed in lung cancer tissues and cell lines and acts as a competing endogenous RNA. Hsa_circ_0012673 combines with miR-320a and thereby regulates LIMK1 expression and promotes the progression of lung cancer [32]. CircNT5E is also significantly upregulated in NSCLC cell lines and lung cancer tissues. CircNT5E promotes the proliferation of cancer cells through spongy miR-134 [33]. However, the role of circANXA2 in lung cancer remains unclear. Further studies on the biological function of circANXA2 in lung adenocarcinoma are needed. In the present study, the expression level of circANXA2 significantly increased in the lung adenocarcinoma tissues, compared with that in the adjacent normal tissues. The downregulation of circANXA2 inhibited the proliferation of lung cancer cells, and the number of circANXA2 cell clones was significantly reduced compared with that in the control group after downregulation. The effect of the downregulation of circANXA2 on cell apoptosis indicated that the cell apoptosis rate was significantly increased in the si-circRNA group. These results indicate that the downregulation of circANXA2 can enhance the apoptotic ability of lung cancer cells. Bioinformatics analysis showed that circANXA2 and miR-33a-5p may interact in complementary regions.

The abnormal expression of miRNAs is involved in tumor progression [34, 35]. By inhibiting the function of target genes, miRNAs play an important role in the coordination of tumor cell proliferation, invasion, invascular perfusion, survival, extravasation, and colonization [36]. The experimental results of Zhang et al. showed that miR-33a expression is significantly lower in human breast cancer tissues compared with that in normal tissues. Moreover, an association was found between decreased miR-33a expression and increased lymph node metastasis in patients with breast cancer [37]. In the present study, we found that compared with normal lung epithelial cells, miR-33a-5p and PDPK1 expression levels were significantly low and high in a variety of lung cancer cells, respectively. The coexpression levels of miR-33a-5p and PDPK1 were negatively correlated in cancer tissues. We predicted the targeting relationship between the two. The results suggest that PDPK1 is a potential target of miR-33a-5p. The results obtained from the luciferase reporter gene supported this prediction, and further experiments demonstrated the regulation of miR-33a-5p on the expression of PDPK1. In addition, the overexpression of miR-33a-5p can reverse the cancer-promoting effect of circANXA2. These results suggest that miR-33a-5p plays a tumor-suppressive role in the malignant progression of lung cancer.

The inhibition of PDPK1 activity with antisense RNA or small molecule inhibitors of PDPK1 induces apoptosis or inhibits the proliferation of cancer cells [38–40]. PDPK1 plays an important role in growth and development [18]. In the present study, PDPK1 expression was upregulated in the lung cancer tissues. Further experiments showed that PDPK1 was the target gene of miR-33a-5p and knocking down circANXA2-reduced PDPK1 expression.

This study has some shortcomings. First, whether an important regulatory mechanism still exists upstream of circANXA2 is unclear. Especially, whether the parent gene of its origin interacts with the regulatory pathway is unknown. As the terminal of the ceRNA axis that we discovered, the downstream signaling pathway regulated by PDPK1 and the mechanism of its influence on the malignant progression of lung adenocarcinoma remain to be clarified. Finally, further study on the role of other miRNAs or RBPs targets of circANXA2 in addition to miR-33a-5p is needed.

## 5. Conclusion

The effects of circANXA2 on the proliferation and apoptosis of lung adenocarcinoma cells and possible mechanisms were explored. The results showed that circANXA2 plays a regulatory role mainly through miR-33a-5p and downstream target gene PDPK1. This finding may provide useful information and a potential target for diagnosis and treatment of lung adenocarcinoma.

## Data Availability

The datasets used and/or analyzed during the current study are available from the corresponding author upon request.
